# Multi-Timescale Conformational Dynamics of the SH3 Domain of CD2-Associated Protein using NMR Spectroscopy and Accelerated Molecular Dynamics**

**DOI:** 10.1002/anie.201202026

**Published:** 2012-05-08

**Authors:** Loïc Salmon, Levi Pierce, Alexander Grimm, Jose-Luis Ortega Roldan, Luca Mollica, Malene Ringkjøbing Jensen, Nico van Nuland, Phineus R L Markwick, J Andrew McCammon, Martin Blackledge

**Affiliations:** Protein Dynamics and Flexibility, Institut de Biologie Structurale Jean-Pierre Ebel, CNRS-CEA-UJF, UMR 507541 rue Jules Horowitz, 38027 Grenoble Cedex (France); Dept of Chemistry and Biochemistry, UCSD, San Diego, Howard Hughes Medical Institute9500 Gilman Dr., La Jolla, CA (USA); Structural Biology Brussels, Vrije Universiteit BrusselPleinlaan, Brussels (Belgium); Dept of Biochemistry, University of OxfordSouth Parks Road, Oxford (UK)

**Keywords:** molecular recognition, NMR spectroscopy, protein dynamics, residual dipolar couplings, spin relaxation

The determination of three-dimensional atomic resolution protein structure underpins our understanding of many biological processes, providing descriptions of the molecular basis of physiologically important interactions between biochemically active molecules. However, a complete understanding of the relationship between biological activity and molecular conformation also requires a description of the thermally accessible potential energy landscape intrinsic to a protein at its physiological temperature.^1^, ^2^

NMR ^15^N and ^13^C spin relaxation experiments are routinely applied for the characterization of rapid motions occurring in proteins on the pico- to nanosecond timescale.^3^ However dynamics occurring on longer timescales, in the nano- to millisecond range, are potentially of greater functional interest, because many biologically important processes, such as enzyme catalysis, signal transduction, ligand binding and allosteric regulation are expected to occur on these timescales.^4^, ^5^ Although longer timescale molecular dynamics (MD) simulations are becoming more accessible,^6^ there is a notable lack of experimental data against which the accuracy of such predictions can be gauged. The precise elucidation of the nature, amplitude and timescale of intrinsic motions occurring in proteins in solution therefore remains a fundamentally important challenge for structural and molecular biologists.

In response to this challenge, there has been considerable activity over the last decade, exploiting the exquisite sensitivity of residual dipolar couplings (RDCs), measured in weakly aligned proteins, to determine the dynamic averaging properties of internuclear bond vectors.^7^–^10^ It has been demonstrated that the measurement of a sufficient number of RDCs in differently aligning media allows the accurate determination of the motional properties of the protein backbone. Two generic approaches to the interpretation of the experimental data can be distinguished—either direct analysis of the RDCs to extract averaged spherical harmonic terms describing the angular averaging of the internuclear bonds,^11^–^14^ or exploiting MD simulation (with or without restraints) to reproduce the motional amplitudes and modes in terms of an explicit conformational ensemble.^15^–^21^

Such RDC-based studies allowed the identification of nano- to millisecond motions that were localized predominantly in the molecular recognition sites of the small proteins ubiquitin (Ub) and GB3.^18^, ^22^, ^25^, ^26^ These discoveries have prompted speculation about the role that intrinsic motions play in molecular recognition, in particular concerning selection of distinct conformers from an existing equilibrium. We note that consideration of the importance of conformational selection for the promiscuity of Ub binding has until now largely neglected the role played by intrinsic dynamics of the partner proteins.

Application of the three dimensional Gaussian Axial Fluctuation (3DGAF) model to Ub^22^ resulted in dynamic modes and amplitudes that were in good agreement with those determined by comparison to restraint-free accelerated MD (AMD) simulation.^23^, ^24^ Nevertheless the nature of slow dynamics occurring in folded proteins, a question of fundamental importance for the understanding of a vast range of biochemical processes, remains the subject of much debate. Until now only two structurally homologous α–β proteins have been studied in sufficient detail to allow a quantitative description of slow dynamics from RDCs, severely limiting our understanding of the general nature of these observations. Here, we analyze the conformational dynamics occurring on timescales from picoseconds to milliseconds in a small β-barrel protein. We simultaneously determine the three-dimensional structure and backbone dynamics of the third SH3 domain of CD2-associated protein (CD2AP)^27^–^30^ (SH3C) directly from RDCs using the 3DGAF approach, and compare the fitted motions to those present in a series of restraint-free AMD simulations. The analyses provide a comprehensive and convergent description of multi-timescale dynamics of this protein, which is a physiological partner of Ub, identifying significant slow conformational fluctuations in the interaction site of the protein.

A total of 1912 RDCs (^1^D_NH_, ^2^D_CHN_ and ^1^D_CaC_) were extracted from ^15^N- and ^13^C-labeled SH3C aligned in 15 alignment media (see Supporting Information). The SECONDA algorithm^31^—a principal component analysis of the RDC covariance matrix—was used to identify data sets that are self-consistent, and therefore show no evidence of perturbing interaction with any medium. Following this, 1358 RDCs from 10 media were retained in the final data set: bicelles,^32^ bicelles doped with CTAB (cetyltrimethylammonium bromide), bicelles doped with SDS (sodium dodecylsulfate), polyacrylamide gel,^33^ PEG (polyethylene glycol)/hexanol,^34^ bacteriophage,^35^ bacteriophage in the presence of high salt, purple membrane^36^ all measured at pH 6.0 and 35 °C. Correlations between ^1^D_NH_ RDCs measured in these media are shown in Figure 1. Three pairs of media resulted in highly correlated alignment, while the seven others present lower, or negligible correlation. The high level of reproduction measured between the three correlated pairs of RDCs testifies to the average precision of the measurement but these data do not provide additional information.

**Figure 1:**
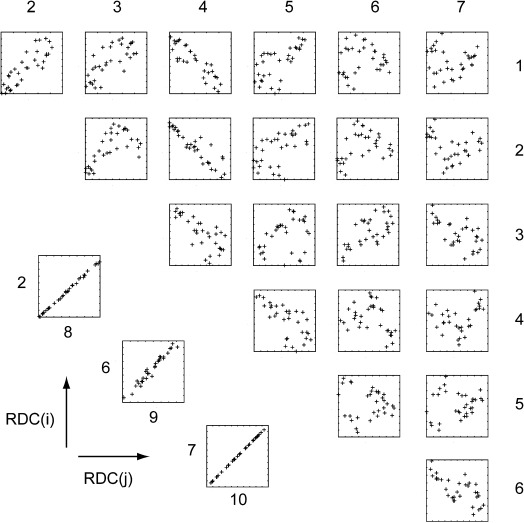
Correlation of the experimental N–^N^H RDCs from SH3C aligned in 10 alignment media (full details in Supporting Information). RDCs are normalized to maximum and minimum experimental values.

The Dynamic-Meccano approach^37^, ^38^ exploits the 1DGAF model combined with the Meccano algorithm,^39^ to determine the average solution structure that explicity accounts for simultaneously determined backbone dynamics. This ab initio structure determination, using only RDCs to construct the peptide chain from GAF-averaged orientations of peptide planes, compares very closely to the nOe and RDC-based NMR structure of SH3C and the X-ray structure of the SH3C from Cin85 (Figure 2).

**Figure 2:**
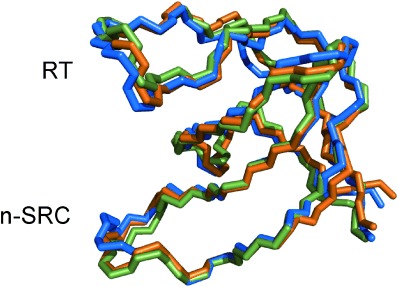
Dynamic Meccano structure of SH3C determined using only RDCs (green) compared to the NMR (orange) structure of SH3C from CD2AP (2kro, backbone rmsd 1.0 Å) and Cin85 (blue) (2ydl, rmsd 0.85 Å), respectively.

The 3DGAF approach that models RDCs in terms of diffusive motions around three orthogonal axes attached to each plane^40^ has been shown to quantitatively describe protein conformational dynamics.^22^ The approach is independent of any structural model, because the average conformation of each peptide plane is determined simultaneously to its dynamic modes. The use of an anisotropic motional model, in combination with RDCs sampling different directions in the peptide plane, allows for an accurate ab initio determination of the alignment tensors.

The 3DGAF analysis was applied as previously described (Supporting Information).^22^ Calculation of randomly selected RDCs (10 %) that were not included in the analysis testifies to the predictive value of the approach (Figure 3a–c) and provides significantly better reproduction of these data than a static structure determination. The 3DGAF motions are dominated by the γ-mode, representing fluctuations about the C^α^–C^α^ axis of each peptide plane of (14.8±6.5)° (see Supporting Information). The N–^N^H bond order parameters determined from the 3DGAF analysis (*S*^2^_3DGAF_) are shown in Figure 3d, in comparison to order parameters derived from ^15^N relaxation (*S*^2^_rel_). The distribution of motions occurring on timescales between nano- and milliseconds is manifest as differences between *S*^2^_3DGAF_ and *S*^2^_rel_, and reveals a similar pattern to that determined in Ub and GB3.^22^, ^26^ Such motions are not ubiquitous throughout the protein. They are indeed found to be negligible in secondary structural elements, whereas in loop regions, in particular the n-SRC (35–43) and RT loops (18–20), significant additional slower motions are apparent. We note that these regions mediate physiological interaction with Ub.^28^

In parallel, we have applied the AMD approach^41^ to the interpretation of the experimental data from SH3C. AMD does not use an experimental pseudo-potential, and is therefore restraint-free. Acceleration is achieved by scaling the potential energy landscape by a constant factor (*α*), for all terms below a given threshold, thereby enhancing the escape rate between low-energy conformational sub-states. On increasing the level of acceleration, the simulation probes more conformational space. Trajectories are re-weighted to obtain a canonical Boltzmann distribution, and a series of short standard MD simulations are seeded from this distribution. The appropriate level of acceleration, and therefore conformational space, is directly estimated by comparing the experimental RDCs to predicted values from ensembles calculated at different levels of acceleration. *R* factors—measuring the quality of the reproduction of each RDC type—are shown in Figure 4 together with the N–^N^H order parameters calculated for each acceleration level (*S*^2^_AMD_). The optimal level of acceleration reproducibly lies around 160 kcal mol^−1^, a value similar to that found for Ub under similar conditions.^24^

**Figure 3:**
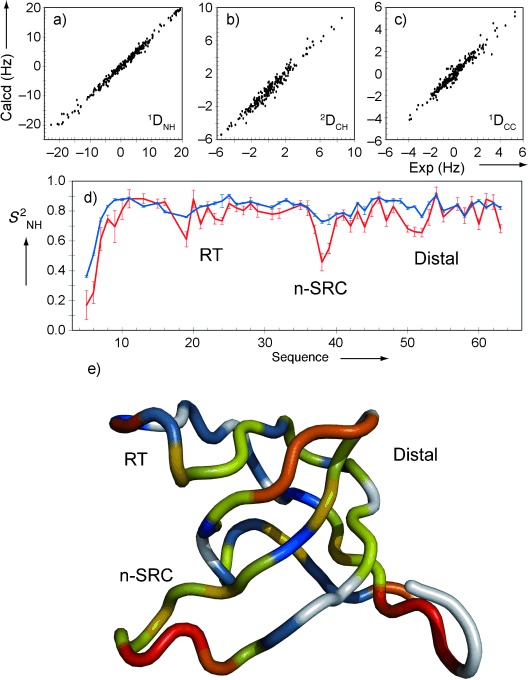
3DGAF analysis of RDC data. a–c) Reproduction of 10 % randomly selected experimental RDCs that were not used in the 3DGAF analysis (10 calculations are shown). d) 3DGAF N–^N^H order parameters (*S*^2^_3DGAF_, red) compared to fast motional order parameters (*S*^2^_rel_, blue). Error bars from noise-based Monte Carlo simulation. e) *S*^2^_3DGAF_ shown on a ribbon representation of the Dynamic Meccano structure (from red (<0.7) through yellow (<0.8) to blue (<0.9); white, no value).

A representative ensemble of structures is shown in Figure 5, together with a comparison of the optimal *S*^2^_AMD_ and *S*^2^_3DGAF_ values. The similarity between the results derived from the two very different approaches, in one case fitting motional modes and amplitudes to the experimental data using mathematical models, and in the other case comparing to restraint free MD simulation, is striking, and substantiates assumptions implicit to both approaches. We have compared *S*^2^_rel_ values with fast motional order parameters derived from the short MD simulations performed from the ensemble of AMD-derived sub-states (Figure 5b). The distribution of fast motions calculated over the entire potential energy surface is quantitatively closer to experimental *S*^2^_rel_ values than any of a series of standard MD simulations starting from the RDC structure (Supporting Information). This provides evidence for the dependence of fast motions on the rugosity of the potential energy landscape sampled on longer timescales.

**Figure 4:**
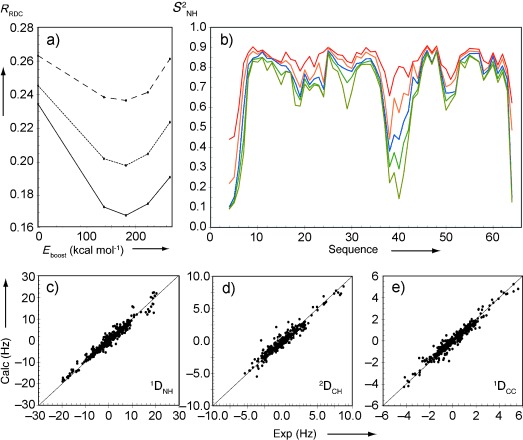
AMD analysis of experimental RDCs. a) Total *Q* values shown for ^1^D_NH_ (solid line), ^2^D_CHN_ (dashed line), and ^1^D_CaC_ (dotted line). b) NH bond order parameters calculated at different acceleration levels (red, standard MD; orange to green, increasing acceleration). c–e) Reproduction of experimental RDCs at the optimal acceleration level.

**Figure 5:**
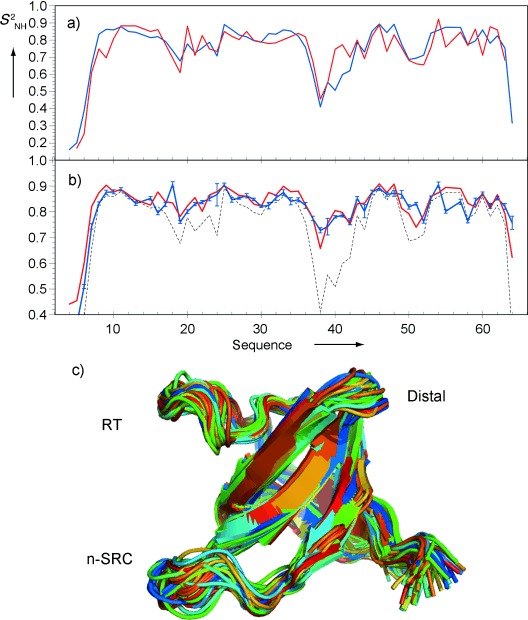
a) AMD-derived (*S*^2^_AMD_, blue) and 3DGAF N–^N^H bond vector order parameters (*S*^2^_3DGAF_, red). b) Fast motional experimental order parameters (*S*^2^_rel_, blue) compared to *S*^2^ values calculated from fast motions occurring in different sub-states sampled by the AMD. *S*^2^_AMD_ values are shown for comparison (dotted lines). c) Representative AMD ensemble.

In conclusion we have measured an extensive set of RDCs in the third SH3 domain of CD2AP, a small β-barrel protein. Independent analyses of RDCs, using analytical fitting of the mean orientation and associated modes and amplitudes of each peptide plane, or comparison with restraint-free AMD simulation, resulted in a comprehensive and remarkably convergent description of multi-timescale dynamics in this protein. Extensive cross-validation procedures were used in both cases to guarantee the self-consistency of each analysis. The similarity of order parameters determined using spin relaxation and RDCs throughout the β-sheet demonstrates the absence of significant conformational fluctuations over timescales spanning up to six orders of magnitude (ns–ms). Importantly these methods both identify large-amplitude slow motions that are localized in the functionally important n-SRC loop that mediates the interaction of SH3C with Ub. These motions are invisible to other biophysical techniques, including NMR spin relaxation. Further studies of the complex between SH3C and Ub will be required to provide additional insight into the role of these slow dynamics, that are localized in the interaction sites of both proteins, in the mechanism of intermolecular recognition.

## Supporting Information

Detailed facts of importance to specialist readers are published as ”Supporting Information”. Such documents are peer-reviewed, but not copy-edited or typeset. They are made available as submitted by the authors.
