# Supplementary material for: Multi-Timescale Conformational Dynamics of the SH3 Domain of CD2-Associated Protein using NMR Spectroscopy and Accelerated Molecular Dynamics
Source: Angew Chem Int Ed Engl. 2012 May 8;51(25):6103–6. doi: 10.1002/anie.201202026 (PMC3541011; doi:10.1002/anie.201202026)
Supplement: Supplementary file 1 [file anie0051-6103-sd1.pdf]

Angewandte Chemie
Eine Zeitschrift der Gesellschaft Deutscher Chemiker

Supporting Information

© Wiley-VCH 2012

69451 Weinheim, Germany

Multi-Timescale Conformational Dynamics of the SH3 Domain of CD2-Associated Protein using NMR Spectroscopy and Accelerated Molecular Dynamics**

*Loïc Salmon, Levi Pierce, Alexander Grimm, Jose-Luis Ortega Roldan, Luca Mollica, Malene Ringkjøbing Jensen, Nico van Nuland, Phineus R. L. Markwick, J. Andrew McCammon, and Martin Blackledge**

anie_201202026_sm_miscellaneous_information.pdf

Supporting Information

S1. Experimental NMR parameters
S2. Alignment media used in the study and SECONDA analysis
S3. α, β and γ GAF amplitudes
S4. Simulation details of the AMD calculation and analysis
S5. Fast motional order parameters calculated from a standard MD simulation in comparison to experimental values.
S6. All experimental RDC values.

S1. NMR Spectroscopy

All measurements were performed at 308 K on Varian NMR Direct-Drive Systems spectrometers at 14.1 T or 18.8 T (corresponding to 600 MHz and 800 MHz ^1H Larmor frequency) equipped with tripe resonance cryoprobes. Relaxation measurements were performed using standard ^1H-^{15}N-HSQC based experiments allowing the successive measurements of ^{15}N-T_1 and T_2 relaxation measurements, $\{^1H\}$-^{15}N nOe experiments[1]. RDCs measurements ($^1D_{NH}$, $^1D_{C'C\alpha}$ and $^1D_{C'H}$) used 3D BEST-type HNCO experiments [2,3] or 2D SOFAST-type experiments for only 15N-labeled samples [4]. NMR spectra were processed in NMRPipe [5] and analyzed using the program Sparky [6].

S2. Alignment media used in the study and SECONDA analysis

RDCs were measured in different alignment media (see below). All samples were prepared in 92% H_2O / 8% D_2O mixture, buffered at pH 6.0 with 50 mM sodium phosphate (NaH_2PO_4/Na_2HPO_4) and 1 mM DTT ((2S,3S)-1,4-bis-sulfanyl-butane-2,3-diol) and recorded at 600MHz. Any deviation from this standard protocol will be explicitly mentioned. All measurements were done in Shigami tubes with 250 µL samples.

Complete sets of $^1D_{NH}$, $^1D_{C'C\alpha}$ and $^1D_{C'H}$ couplings were measured in the following alignment media:

1. A 5% (w/w) bicelle mixture of ditetradecyl-PC (1,2-di-O-tetradecyl-sn-glycero-3-phosphocholine) and dihexyl-PC (1,2-di-O-hexyl-sn-glycero-3-phosphocholine) in a 3:1 ratio [7].
2. A 5% bicelles mixture doped with CTAB (hexadecyl-trimethyl-ammonium bromide) in a ratio ditetradecyl-PC:dihexyl-PC:CTAB 30:10:1 [7,8,9] and measured at 800 MHz.
3. A 5% bicelles mixture doped with CTAB in a ratio ditetradecyl-PC:dihexyl-PC:CTAB 30:10:0.5.
4. A 5% bicelles mixture charged with SDS (sodium dodecyl-sulfate) [8,9] in the following ratio ditetradecyl-PC:dihexyl-PC:SDS 30:10:1.
5. A 5% penta-ethyleneglycol monododecyl ether hexanol mixture [10].
6. A 9 mg/mL bacteriophages Pf1 mixture at pH 6.5 and measured at 800 MHz [11].
7. A 15 mg/mL bacteriophages Pf1 mixture at pH 6.5 at 150 mM NaCl concentration.
8. A 13.1 mg/mL bacteriophages Pf1 mixture at pH 6.5 at 260 mM NaCl concentration and measured at 800 MHz.
9. Compressed 7% polyacrylamide gel [12].

$^1D_{NH}$ couplings were also measured in:

10. A 5% bicelles mixture doped with CTAB in a ratio ditetradecyl-PC: dihexyl-PC:CTAB 30:10:0.1 and measured at 800 MHz.
11. A 5% bicelles mixture doped with CTAB in a ratio ditetradecyl-PC: dihexyl-PC:CTAB 30:10:0.2 and measured at 800 MHz.
12. A 5% bicelles mixture doped with CTAB in a ratio ditetradecyl-PC: dihexyl-PC:CTAB 30:10:1.5 and measured at 800 MHz.
13. A 5% bicelles mixture doped with SDS in a ratio ditetradecyl-PC: dihexyl-PC:SDS 30:10:0.5 and measured at 800 MHz.
14. A 5% bicelles mixture doped with SDS in a ratio ditetradecyl-PC: dihexyl-PC:SDS 30:10:1 and measured at 800 MHz.
15. Purple membrane fragments mixture with 150 mM NaCl. Purple Membranes were added until a reasonable range of couplings were reached (±8 Hz) [13,14].

A Self-Consistency analysis based on the Seconda approach [15,16] was performed using the $^1D_{NH}$ couplings measured in the 15 alignment media. By repeating the analysis for all possible combinations of 14, 13, 12, 11, 10, 9 or 8 dataset sub-ensembles, a compromise between the self-consistency and the number of remaining datasets was found by selecting the 10 datasets made of the following media: 1, 3, 4, 5, 6, 7, 8, 9, 12 and 15. For this selected sub-ensemble the ratio between the fifth and the sixth eigenvalues obtained through the diagonalization of the weighted covariance matrix build up with the experimental RDCs that characterize the self consistency of the dataset was found to be 7.9.

1. N. A. Farrow, R. Muhandiram, A. U. Singer, S. M. Pascal, C. M. Kay, G. Gish, S. E. Shoelson, T. Pawson, J. D. Forman-Kay, and L. E. Kay. Biochemistry, 33(19):5984–6003, 1994.
2. P. Schanda, H. V. Melckebeke, and B. Brutscher. J Am Chem Soc, 128(28):9042–9043, 2006.
3. E. Lescop, P. Schanda, and B. Brutscher. J Magn Res, 187(1):163–169, 2007.
4. P. Schanda and B. Brutscher. J Am Chem Soc, 127(22):8014–8015, 2005.
5. F. Delaglio, S. Grzesiek, G. W. Vuister, G. Zhu, J. Pfeifer, and A. Bax. J Biomol NMR, 6(3):277–93, 1995.
6. T. Goddard and D. Kneller. University of California, 2003.
7. M. Ottiger and A. Bax. J Biomol NMR, 13(2):187–191, 1999.
8. J. A. Losonczi and J. H. Prestegard. J Biomol NMR, 12(3):447–51, 1998.
9. B. Ramirez and A. Bax. J Am Chem Soc, 120(35):9106–9107, 1998.

10. M. Rückert and G. Otting. J Am Chem Soc, 122 (32):7793–7797, 2000.
11. H.-J. Sass, G. Musco, S. Stahl, P. T. Wingfield, and S. Grzesiek. J Biomol NMR, 18(4):303–309, 2000.
12. Hansen, M.; Mueller, L.; Pardi, A. *Nat. Struct. Biol.* **1998**, *5*, 1065-1074
13. B. Koenig, J. Hu, M. Ottiger, S. Bose, R. Hendler, and A. Bax. J Am Chem Soc, 121(6):1385–1386, 1999.
14. B. W. Koenig, D. C. Mitchell, S. König, S. Grzesiek, B. J. Litman, and A. Bax. J Biomol NMR, 16(2):121–5, 2000.
15. J.-C. Hus and R. Brüschweiler. J Biomol NMR, 24 (2):123–32, 2002
16. J.-C. Hus, W. Peti, C. Griesinger, and R. Brüschweiler. J Am Chem Soc, 125(19):5596–5597, 2003.

S3. α, β and γ GAF amplitudes (in degrees)

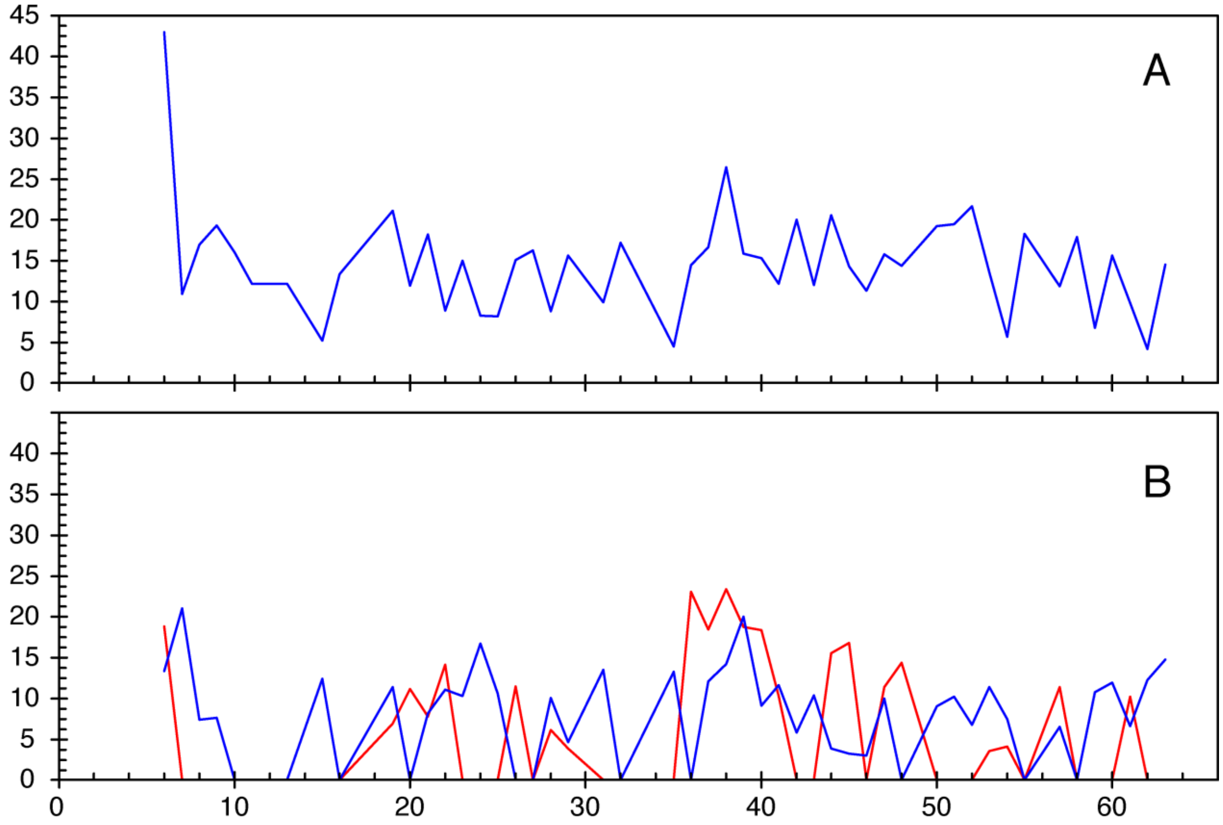

GAF motional amplitudes from the 3DGAF analysis of RDC data form SH3C.
A – amplitude of gamma motions (σ_γ in degrees)
B – amplitude of beta (red) and alpha motions (σ_α and σ_β in degrees)

S4. Simulation details of the AMD calculation and analysis

(1) Accelerated Molecular Dynamics (AMD):

The details of the accelerated molecular dynamics (AMD) protocol have been discussed previously in the literature [P1,P2] and only a brief summary is provided here. In the AMD approach a reference or 'boost energy', E_b, is defined which lies above the minimum of the potential energy surface. At each step in simulation, if the instantaneous potential energy, V(r), lies below the boost energy a continuous, non-negative bias potential,ΔV(r), is added to the actual potential. If the potential energy is greater than the boost energy, it remains unaltered. The application of the bias potential results in a raising and flattening of the potential energy surface (PES), decreasing the magnitude of the energy barriers and thereby accelerating the exchange between low energy conformational states, whilst still maintaining the essential details of the potential energy landscape. Explicitly, the modified potential, V*(r), on which the system evolves during an AMD simulation is given by:

$$V^*(\vec{r}) = V(\vec{r})$$
$$V^*(\vec{r}) = V(\vec{r}) + \Delta V(\vec{r})$$

and the bias potential, ΔV(r), is defined as:

$$\Delta V(\vec{r}) = \frac{(E_b - V(\vec{r}))^2}{\alpha + (E_b - V(\vec{r}))}$$

The extent of acceleration (ie. how aggressively the conformational space is sampled) is determined by the choice of the boost energy, E_b, and the acceleration parameter, α. Conformational space sampling can be enhanced by either increasing the boost energy, or decreasing α. During the course of the AMD simulation, if the potential energy is modified, the forces on the atoms are recalculated for the modified potential and the use of the bias potential defined above ensures that the derivative of the modified potential will not become discontinuous at points where $V(r)=E_b$.

One of the favorable characteristics of the AMD approach is that it yields a canonical average of an ensemble, so that thermodynamic and other equilibrium properties can be accurately determined. The corrected canonical ensemble average of the system is obtained by re-weighting each point in the configuration space on the modified potential by the strength of the Boltzmann factor of the bias energy, *exp*[$\beta\Delta V(r_{t(i)})$] at that particular point.

Accelerated molecular dynamics is an extended biased potential MD approach that allows for the efficient study of bio-molecular systems up to time-scales several orders of magnitude greater than those accessible using standard classical MD approaches, whilst still maintaining a fully atomistic representation of the system. AMD has already been employed with great success to study the dynamics and conformational behavior of a variety of bio-molecular systems including poly-peptides, folded and natively unstructured proteins. [P2]

(2) Calculation of N-H^N Residual Dipolar Couplings:

The general protocol employed here to calculate residual dipolar couplings (RDCs) follows along lines similar to those detailed in previous studies on ubiquitin[P3] and IκBα[P4]. Atomic coordinates for SH3 were obtained from the Dynamic Meccano structure determined using RDCs as described in the main manuscript and the system was placed in a periodically repeating box with ~10000 water molecules and three Na+ counter-ions. Initially a set of eight standard classical MD (CMD) simulations were performed. For each of these simulations, a different random seed generator for the Maxwellian distribution of atomic velocities was employed and, after standard energy minimization and equilibration procedures, a 10-ns production run MD simulation was performed under periodic boundary conditions with a time-step of 2-fs. Bonds involving protons were constrained using the SHAKE algorithm. Electrostatic interactions were treated using the Particle Mesh Ewald (PME) method with a direct space sum limit of 10-A. The ff99SB force-field was used for the solute residues and the TIP3P water force-field was employed for the solvent molecules. These initial eight 10-ns CMD simulations acted as a control set and were used as the starting point for the AMD simulations. These simulations also provided the average (unbiased) dihedral angle energy, $\langle V_0(dih)\rangle$ used to define the acceleration parameters in the AMD simulations described below.

A series of eight torsional AMD simulations were performed for 5,000,000 steps (the equivalent of 10-ns) at increasing levels of acceleration. For the torsional acceleration, the acceleration parameter, α(dih) was fixed at 45-kcal/mol and the torsional boost energy, E_b(dih) for the four acceleration levels was set at 135, 180, 225 and 270-kcal/mol above the average dihedral angle energy, $\langle V_0(dih)\rangle$ estimated from the unbiased 10-ns CMD simulations. The physical conditions, force-fields and all other simulation parameters employed in the AMD simulations were identical to those described for the standard CMD trajectories. All MD and AMD simulations were performed using an in-house modified version of the AMBER 10 code.[P5]

For each AMD simulation, a corrected canonical ensemble was obtained by performing the Boltzmann free energy re-weighting protocol described above using the bias potential block averaging method [P2] to remove statistical noise errors. In this way, eight representative free-energy weighted molecular ensembles were generated at each of the four acceleration levels, along with the eight unbiased 10-ns CMD simulations. For each free energy weighted molecular ensemble, an RMSD-based clustering algorithm was employed to identify low energy conformational states. A series of short 3-ns standard MD simulations were then seeded from the AMD simulations to sample the low energy states. The initial 0.5-ns were discarded, and a MM/PBSA[P6] analysis on the resulting MD simulations was used to confirm the approximate AMD free energy

weighting protocol. Using these approximate free energies, a set of large (free energy weighted) structural ensembles was generated from the seeded MD simulations for each acceleration level. A Singular Value Decomposition (SVD) analysis [P7] was performed to determine the optimal alignment tensor for each free-energy weighted ensemble, and N-H^N RDCs were then calculated. The resulting RDC values for each free energy weighted ensemble associated with a given acceleration level were then averaged. The agreement between the theoretical and experimental RDCs was monitored using the well-known R-factor:

$$R_{factor} = \sqrt{\frac{\sum_{i=1}^{N}(X(i)_{theory} - X(i)_{\exp})^2}{2\sum_{i=1}^{N}(X(i)_{\exp})^2}}$$

where $X(i)_{theory}$ and $X(i)_{exp}$ are the theoretically determined and experimental observables respectively. The "optimal" torsional acceleration level (and hence the "optimal" conformational space sampling) for the reproduction of the experimental RDCs was found to be at [E_b(dih)-V_0(dih)=180-kcal/mol, α(dih)= 45-kcal/mol].

(3) Calculation of Order Parameters:

The internal dynamics present in the different MD and AMD simulations of SH3 were monitored by calculating the order parameters (S^2) relevant for the Lipari-Szabo type analysis [P8] of ^{15}N auto-relaxation data and for the RDC-derived order parameters respectively. In all cases, molecular ensembles generated from the standard CMD simulations and the free-energy weighted molecular ensembles generated from the AMD trajectories were superposed onto the heavy backbone atoms of all residues [5-60] for the appropriate average structure. Order parameters were calculated as:

$$S^2 = \frac{1}{2}\left[3\sum_{i=1}^{3}\sum_{j=1}^{3} < \mu_i \mu_j >^2 - 1\right]$$

where μ_i are the Cartesian coordinates of the normalized inter-nuclear vector of interest.

[P1] D. Hamelberg, J. Mongan and J.A. McCammon, *J. Chem. Phys.* **2004**, *120*, 11919-11929.
[P2] P.R.L. Markwick and J.A. McCammon, *Phys. Chem. Chem. Phys.* **2011**, *13*, 20053-20065.
[P3] P.R.L. Markwick, G. Bouvignies, L. Salmon, J.A. McCammon and M. Blackledge, *J. Am. Chem. Soc.* **2009**, *131*, 16968-16975.
[P4] C.F. Cervantes, P.R.L. Markwick, S.-C. Sue, J.A. McCammon, H.J. Dyson and E.A. Komives, *Biochemistry*, **2009**, *48*, 8023-8031.
[P5] D.A. Case et al. **2008**, AMBER10, University of California, San Francisco.
[P6] I. Massova and P.A. Kollman, *J. Am. Chem. Soc.* **1999**, *121*, 8133-8143.
[P7] J.A. Losonczi, M. Andrec, M.W. Fischer and J.H. Prestegard, *J. Magn. Reson.* **1999**, *138*, 334-342; S.A. Showalter and R. Brueschweiler, *J. Am. Chem. Soc.* **2007**, *129*, 4158-4159.
[P8] G. Lipari and A. Szabo, *J. Am. Chem. Soc.* **1982**, *104*, 4546-4559.

S5. Fast motional order parameters calculated from a standard MD simulation in comparison to experimental values.

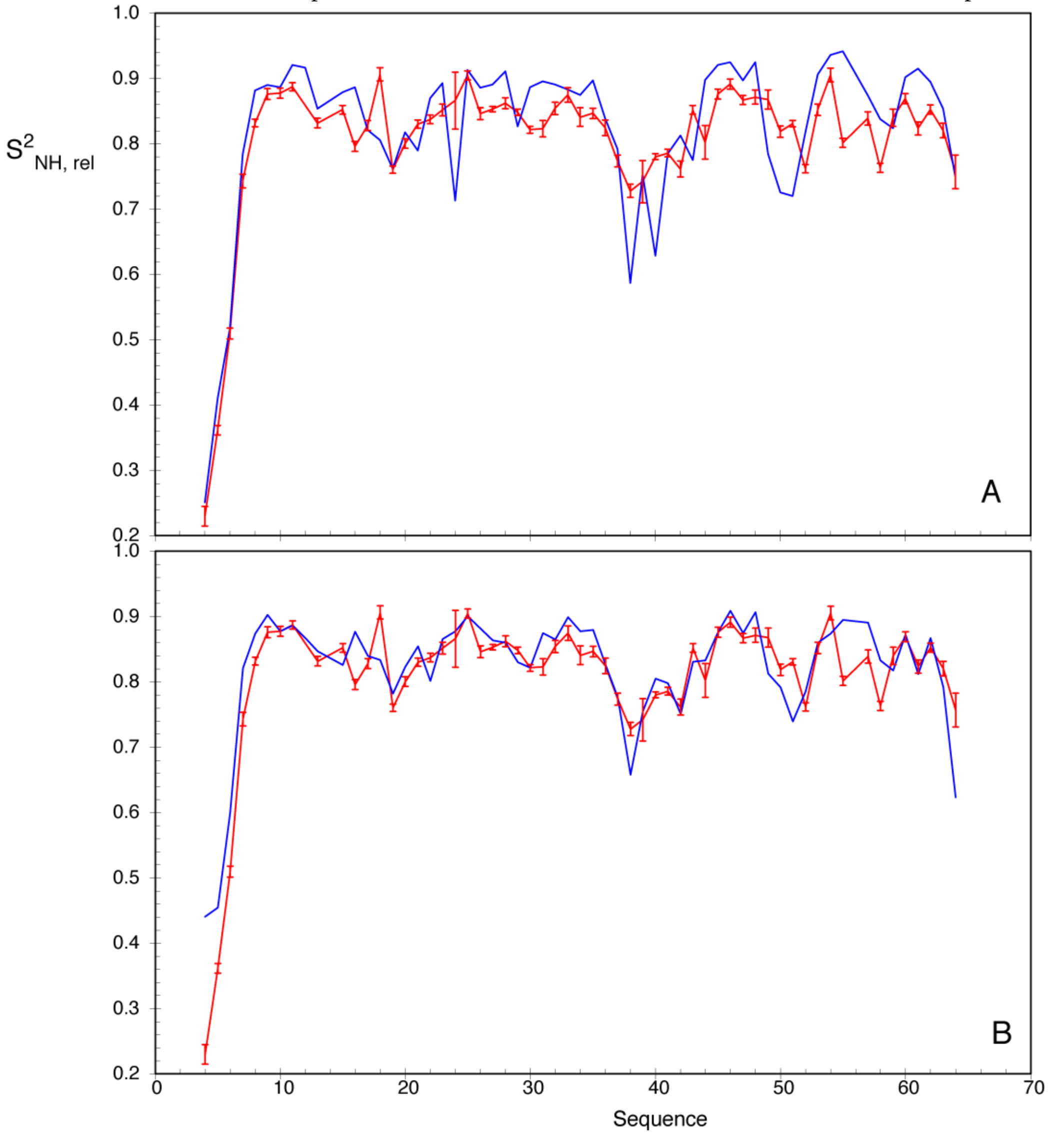

A: Order parameters derived from standard MD simulation of 5ns of SH3C using the Gromacs program with the AMBER99SB force field (blue) compared to fast motional order parameters derived from spin relaxation (red). Shown is the best reproduction provided by one of ten different MD simulations.
B: Order parameters derived from standard 5ns MD simulations from different conformations derived from the AMD calculation of SH3C (blue) compared to fast motional order parameters derived from spin relaxation (red).

S6. All experimental RDC values (in Hz).

Alignment medium, residue number1, atom1, residue number2, atom2, value, uncertainty

0. 6 N 6 HN -2.703 0.573
0. 7 N 7 HN 1.089 0.573
0. 8 N 8 HN 3.721 0.573
0. 9 N 9 HN -2.679 0.573
0. 11 N 11 HN -10.446 0.573
0. 12 N 12 HN -0.865 0.573
0. 13 N 13 HN -1.489 0.573
0. 15 N 15 HN -8.126 0.573
0. 16 N 16 HN -9.660 0.573
0. 17 N 17 HN -6.331 0.573
0. 18 N 18 HN 4.347 0.573
0. 19 N 19 HN 7.315 0.573
0. 21 N 21 HN 0.249 0.573
0. 22 N 22 HN 9.680 0.573
0. 23 N 23 HN -3.920 0.573
0. 24 N 24 HN -7.770 0.573
0. 25 N 25 HN -6.023 0.573
0. 26 N 26 HN -3.696 0.573
0. 27 N 27 HN 4.259 0.573
0. 28 N 28 HN -5.965 0.573
0. 29 N 29 HN -2.833 0.573
0. 30 N 30 HN -7.993 0.573
0. 31 N 31 HN -6.743 0.573
0. 32 N 32 HN 3.346 0.573
0. 33 N 33 HN 7.842 0.573
0. 34 N 34 HN 5.983 0.573
0. 35 N 35 HN 5.701 0.573
0. 36 N 36 HN 7.998 0.573
0. 37 N 37 HN -7.094 0.573
0. 38 N 38 HN -6.337 0.573
0. 40 N 40 HN -3.173 0.573
0. 41 N 41 HN -1.658 0.573
0. 42 N 42 HN 7.142 0.573
0. 43 N 43 HN 0.361 0.573
0. 44 N 44 HN 4.075 0.573
0. 45 N 45 HN 8.206 0.573
0. 46 N 46 HN 10.707 0.573
0. 47 N 47 HN 8.502 0.573
0. 48 N 48 HN 4.994 0.573
0. 49 N 49 HN 8.985 0.573
0. 50 N 50 HN 4.663 0.573
0. 51 N 51 HN 0.219 0.573
0. 52 N 52 HN 0.873 0.573
0. 53 N 53 HN 8.413 0.573
0. 54 N 54 HN 9.250 0.573
0. 55 N 55 HN 4.857 0.573
0. 57 N 57 HN -0.131 0.573
0. 58 N 58 HN 6.878 0.573
0. 59 N 59 HN -1.376 0.573
0. 60 N 60 HN -4.368 0.573
0. 61 N 61 HN -6.939 0.573
0. 62 N 62 HN -8.900 0.573
0. 63 N 63 HN -2.931 0.573
0. 64 N 64 HN -0.273 0.573
0. 6 C 7 HN -1.132 0.346
0. 7 C 8 HN 0.608 0.346
0. 8 C 9 HN -2.928 0.346
0. 9 C 10 HN 2.730 0.346
0. 10 C 11 HN 1.082 0.346
0. 12 C 13 HN -0.252 0.346
0. 14 C 15 HN 2.854 0.346
0. 15 C 16 HN -0.592 0.346
0. 16 C 17 HN 2.826 0.346
0. 17 C 18 HN 0.882 0.346
0. 18 C 19 HN -2.498 0.346
0. 19 C 20 HN -0.388 0.346
0. 20 C 21 HN 2.602 0.346
0. 21 C 22 HN -2.006 0.346
0. 22 C 23 HN -1.300 0.346
0. 23 C 24 HN 3.088 0.346
0. 24 C 25 HN 2.576 0.346
0. 25 C 26 HN -0.044 0.346
0. 26 C 27 HN 0.252 0.346
0. 27 C 28 HN -0.850 0.346

0. 28 C 29 HN -1.144 0.346
0. 29 C 30 HN 3.836 0.346
0. 30 C 31 HN 3.066 0.346
0. 31 C 32 HN 1.456 0.346
0. 32 C 33 HN -2.314 0.346
0. 33 C 34 HN -3.076 0.346
0. 34 C 35 HN -1.366 0.346
0. 35 C 36 HN -1.596 0.346
0. 36 C 37 HN 1.870 0.346
0. 37 C 38 HN -0.264 0.346
0. 38 C 39 HN 0.308 0.346
0. 39 C 40 HN 0.268 0.346
0. 40 C 41 HN 2.480 0.346
0. 41 C 42 HN -3.278 0.346
0. 42 C 43 HN 0.168 0.346
0. 43 C 44 HN 1.062 0.346
0. 44 C 45 HN -2.316 0.346
0. 46 C 47 HN -2.562 0.346
0. 47 C 48 HN -2.650 0.346
0. 50 C 51 HN 2.374 0.346
0. 51 C 52 HN 0.230 0.346
0. 52 C 53 HN 0.494 0.346
0. 53 C 54 HN -2.540 0.346
0. 54 C 55 HN -0.760 0.346
0. 56 C 57 HN 1.214 0.346
0. 57 C 58 HN -1.610 0.346
0. 58 C 59 HN 1.288 0.346
0. 59 C 60 HN -0.564 0.346
0. 60 C 61 HN 1.228 0.346
0. 61 C 62 HN 0.846 0.346
0. 62 C 63 HN 3.484 0.346
0. 63 C 64 HN -0.126 0.346
0. 6 CA 6 C 1.396 0.217
0. 7 CA 7 C -0.974 0.217
0. 8 CA 8 C 1.830 0.217
0. 9 CA 9 C -0.612 0.217
0. 11 CA 11 C 0.390 0.217
0. 14 CA 14 C 0.428 0.217
0. 15 CA 15 C 0.326 0.217
0. 16 CA 16 C 0.394 0.217
0. 17 CA 17 C -2.008 0.217
0. 18 CA 18 C 0.594 0.217
0. 19 CA 19 C 0.402 0.217
0. 20 CA 20 C -1.820 0.217
0. 21 CA 21 C -0.248 0.217
0. 22 CA 22 C 0.612 0.217
0. 23 CA 23 C 0.044 0.217
0. 24 CA 24 C -0.864 0.217
0. 25 CA 25 C 0.444 0.217
0. 26 CA 26 C -0.940 0.217
0. 27 CA 27 C -0.084 0.217
0. 28 CA 28 C 1.432 0.217
0. 30 CA 30 C -0.040 0.217
0. 31 CA 31 C -0.938 0.217
0. 32 CA 32 C 0.680 0.217
0. 33 CA 33 C 0.424 0.217
0. 34 CA 34 C 0.975 0.217
0. 35 CA 35 C 1.128 0.217
0. 36 CA 36 C -0.240 0.217
0. 37 CA 37 C -0.526 0.217
0. 38 CA 38 C -0.438 0.217
0. 39 CA 39 C 0.162 0.217
0. 40 CA 40 C -0.022 0.217
0. 41 CA 41 C -0.102 0.217
0. 42 CA 42 C 1.850 0.217
0. 43 CA 43 C -0.634 0.217
0. 44 CA 44 C 1.512 0.217
0. 45 CA 45 C 0.100 0.217
0. 46 CA 46 C 1.250 0.217
0. 47 CA 47 C 0.450 0.217
0. 48 CA 48 C 1.288 0.217
0. 49 CA 49 C -0.556 0.217
0. 50 CA 50 C -0.858 0.217
0. 51 CA 51 C 1.600 0.217
0. 52 CA 52 C -0.038 0.217
0. 53 CA 53 C 1.366 0.217
0. 54 CA 54 C -0.214 0.217
0. 56 CA 56 C -1.968 0.217

0. 57 CA 57 C 0.586 0.217
0. 58 CA 58 C 0.834 0.217
0. 59 CA 59 C -0.466 0.217
0. 60 CA 60 C -1.274 0.217
0. 61 CA 61 C 0.590 0.217
0. 62 CA 62 C -0.992 0.217
2. 7 N 7 HN -3.403 0.539
2. 8 N 8 HN 4.574 0.539
2. 9 N 9 HN -0.060 0.539
2. 10 N 10 HN -5.969 0.539
2. 13 N 13 HN 3.050 0.539
2. 15 N 15 HN -19.616 0.539
2. 16 N 16 HN -19.750 0.539
2. 17 N 17 HN -17.784 0.539
2. 18 N 18 HN 17.256 0.539
2. 19 N 19 HN 3.355 0.539
2. 20 N 20 HN 0.720 0.539
2. 22 N 22 HN 18.124 0.539
2. 23 N 23 HN 3.052 0.539
2. 24 N 24 HN -16.442 0.539
2. 25 N 25 HN -17.298 0.539
2. 26 N 26 HN -13.870 0.539
2. 27 N 27 HN 18.803 0.539
2. 28 N 28 HN -4.165 0.539
2. 30 N 30 HN -7.738 0.539
2. 31 N 31 HN -7.515 0.539
2. 32 N 32 HN 7.975 0.539
2. 33 N 33 HN 1.686 0.539
2. 35 N 35 HN 19.212 0.539
2. 37 N 37 HN -15.294 0.539
2. 38 N 38 HN -9.969 0.539
2. 39 N 39 HN 3.435 0.539
2. 40 N 40 HN -13.491 0.539
2. 41 N 41 HN -9.909 0.539
2. 43 N 43 HN 10.538 0.539
2. 44 N 44 HN 18.946 0.539
2. 45 N 45 HN 20.380 0.539
2. 46 N 46 HN 13.734 0.539
2. 47 N 47 HN 8.954 0.539
2. 48 N 48 HN 0.764 0.539
2. 49 N 49 HN 7.698 0.539
2. 50 N 50 HN 10.795 0.539
2. 51 N 51 HN -1.057 0.539
2. 52 N 52 HN -1.267 0.539
2. 53 N 53 HN 7.965 0.539
2. 54 N 54 HN 8.954 0.539
2. 55 N 55 HN 19.333 0.539
2. 57 N 57 HN -6.941 0.539
2. 58 N 58 HN -1.300 0.539
2. 59 N 59 HN -0.488 0.539
2. 60 N 60 HN -2.010 0.539
2. 61 N 61 HN -6.684 0.539
2. 62 N 62 HN -13.147 0.539
2. 63 N 63 HN -5.094 0.539
2. 6 C 7 HN 1.868 0.492
2. 7 C 8 HN 1.108 0.492
2. 9 C 10 HN 3.570 0.492
2. 11 C 12 HN 5.182 0.492
2. 12 C 13 HN 1.398 0.492
2. 14 C 15 HN 4.994 0.492
2. 15 C 16 HN 3.018 0.492
2. 16 C 17 HN 5.606 0.492
2. 17 C 18 HN -1.408 0.492
2. 18 C 19 HN -1.858 0.492
2. 19 C 20 HN 1.562 0.492
2. 20 C 21 HN 5.292 0.492
2. 22 C 23 HN -2.700 0.492
2. 23 C 24 HN 5.098 0.492
2. 24 C 25 HN 5.366 0.492
2. 26 C 27 HN -0.618 0.492
2. 27 C 28 HN -5.200 0.492
2. 28 C 29 HN 2.496 0.492
2. 29 C 30 HN 6.566 0.492
2. 30 C 31 HN 2.886 0.492
2. 31 C 32 HN 2.736 0.492
2. 32 C 33 HN -4.174 0.492
2. 33 C 34 HN -1.926 0.492
2. 34 C 35 HN -0.866 0.492

2. 36 C 37 HN 2.780 0.492
2. 37 C 38 HN -0.334 0.492
2. 38 C 39 HN -1.162 0.492
2. 39 C 40 HN 0.498 0.492
2. 40 C 41 HN 5.480 0.492
2. 41 C 42 HN -1.358 0.492
2. 42 C 43 HN -1.712 0.492
2. 43 C 44 HN -1.248 0.492
2. 44 C 45 HN -3.396 0.492
2. 46 C 47 HN 1.198 0.492
2. 47 C 48 HN -3.950 0.492
2. 50 C 51 HN 2.624 0.492
2. 51 C 52 HN 0.950 0.492
2. 52 C 53 HN -0.136 0.492
2. 53 C 54 HN 1.040 0.492
2. 54 C 55 HN -3.690 0.492
2. 56 C 57 HN -0.986 0.492
2. 57 C 58 HN -0.160 0.492
2. 60 C 61 HN 4.618 0.492
2. 61 C 62 HN -2.534 0.492
2. 62 C 63 HN 4.984 0.492
2. 63 C 64 HN 0.844 0.492
2. 8 CA 8 C 3.110 0.272
2. 9 CA 9 C -3.432 0.272
2. 11 CA 11 C 0.830 0.272
2. 12 CA 12 C -4.118 0.272
2. 14 CA 14 C 1.278 0.272
2. 15 CA 15 C 0.456 0.272
2. 16 CA 16 C 0.234 0.272
2. 17 CA 17 C -0.808 0.272
2. 18 CA 18 C 0.664 0.272
2. 19 CA 19 C 2.232 0.272
2. 20 CA 20 C -3.880 0.272
2. 21 CA 21 C 0.672 0.272
2. 22 CA 22 C 1.842 0.272
2. 23 CA 23 C -1.246 0.272
2. 24 CA 24 C 1.176 0.272
2. 25 CA 25 C -0.236 0.272
2. 26 CA 26 C -2.560 0.272
2. 27 CA 27 C 2.886 0.272
2. 28 CA 28 C 2.152 0.272
2. 29 CA 29 C -3.824 0.272
2. 30 CA 30 C 0.400 0.272
2. 31 CA 31 C -4.178 0.272
2. 32 CA 32 C 1.910 0.272
2. 33 CA 33 C -0.766 0.272
2. 34 CA 34 C 0.725 0.272
2. 35 CA 35 C 2.518 0.272
2. 36 CA 36 C 1.490 0.272
2. 37 CA 37 C -0.766 0.272
2. 38 CA 38 C 1.622 0.272
2. 39 CA 39 C 1.072 0.272
2. 40 CA 40 C 0.538 0.272
2. 41 CA 41 C -2.432 0.272
2. 42 CA 42 C 1.630 0.272
2. 43 CA 43 C 0.546 0.272
2. 44 CA 44 C 1.642 0.272
2. 45 CA 45 C 2.630 0.272
2. 46 CA 46 C 1.030 0.272
2. 47 CA 47 C -0.680 0.272
2. 48 CA 48 C 0.348 0.272
2. 49 CA 49 C 0.104 0.272
2. 50 CA 50 C 2.032 0.272
2. 51 CA 51 C -0.010 0.272
2. 52 CA 52 C 2.392 0.272
2. 53 CA 53 C 1.726 0.272
2. 54 CA 54 C -1.294 0.272
2. 56 CA 56 C -1.648 0.272
2. 57 CA 57 C 0.796 0.272
2. 58 CA 58 C -0.906 0.272
2. 59 CA 59 C 2.144 0.272
2. 60 CA 60 C -3.214 0.272
2. 61 CA 61 C 1.770 0.272
2. 62 CA 62 C 1.248 0.272
2. 63 CA 63 C -2.400 0.272
3. 4 N 4 HN -0.088 0.486
3. 5 N 5 HN -0.953 0.486
3. 7 N 7 HN 5.787 0.486

3. 8 N 8 HN 7.426 0.486
3. 11 N 11 HN -4.025 0.486
3. 12 N 12 HN 1.940 0.486
3. 13 N 13 HN 0.052 0.486
3. 15 N 15 HN -6.418 0.486
3. 16 N 16 HN -7.626 0.486
3. 17 N 17 HN -5.783 0.486
3. 18 N 18 HN 3.857 0.486
3. 19 N 19 HN 4.968 0.486
3. 20 N 20 HN 6.135 0.486
3. 21 N 21 HN -4.873 0.486
3. 22 N 22 HN 2.937 0.486
3. 23 N 23 HN 1.145 0.486
3. 24 N 24 HN -6.416 0.486
3. 25 N 25 HN -8.116 0.486
3. 26 N 26 HN -8.015 0.486
3. 27 N 27 HN -2.390 0.486
3. 28 N 28 HN -1.366 0.486
3. 29 N 29 HN -4.849 0.486
3. 30 N 30 HN -1.420 0.486
3. 31 N 31 HN 2.420 0.486
3. 32 N 32 HN 6.069 0.486
3. 33 N 33 HN 5.791 0.486
3. 35 N 35 HN -2.670 0.486
3. 36 N 36 HN -2.203 0.486
3. 37 N 37 HN -6.896 0.486
3. 38 N 38 HN -2.174 0.486
3. 39 N 39 HN 5.995 0.486
3. 40 N 40 HN -2.301 0.486
3. 41 N 41 HN -0.675 0.486
3. 42 N 42 HN -1.687 0.486
3. 43 N 43 HN 0.698 0.486
3. 44 N 44 HN 0.423 0.486
3. 45 N 45 HN 1.680 0.486
3. 46 N 46 HN 3.112 0.486
3. 47 N 47 HN 6.504 0.486
3. 48 N 48 HN 8.040 0.486
3. 49 N 49 HN 8.148 0.486
3. 50 N 50 HN 6.115 0.486
3. 51 N 51 HN 5.202 0.486
3. 52 N 52 HN 6.702 0.486
3. 53 N 53 HN 6.221 0.486
3. 54 N 54 HN 6.764 0.486
3. 57 N 57 HN 0.551 0.486
3. 58 N 58 HN 2.883 0.486
3. 59 N 59 HN 3.728 0.486
3. 60 N 60 HN -1.175 0.486
3. 61 N 61 HN -2.016 0.486
3. 62 N 62 HN -4.000 0.486
3. 63 N 63 HN 5.072 0.486
3. 64 N 64 HN -0.239 0.486
3. 3 C 4 HN -1.525 0.551
3. 4 C 5 HN 0.564 0.551
3. 8 C 9 HN -1.628 0.551
3. 10 C 11 HN -0.328 0.551
3. 11 C 12 HN 0.862 0.551
3. 12 C 13 HN -2.042 0.551
3. 14 C 15 HN 1.164 0.551
3. 15 C 16 HN 2.288 0.551
3. 16 C 17 HN 2.926 0.551
3. 17 C 18 HN 0.082 0.551
3. 18 C 19 HN 0.892 0.551
3. 19 C 20 HN -2.898 0.551
3. 20 C 21 HN 0.442 0.551
3. 21 C 22 HN 1.604 0.551
3. 22 C 23 HN -2.320 0.551
3. 23 C 24 HN 1.288 0.551
3. 24 C 25 HN 0.386 0.551
3. 25 C 26 HN 1.936 0.551
3. 26 C 27 HN 0.482 0.551
3. 27 C 28 HN -1.160 0.551
3. 29 C 30 HN 2.546 0.551
3. 31 C 32 HN -0.884 0.551
3. 32 C 33 HN -2.564 0.551
3. 33 C 34 HN 0.454 0.551
3. 35 C 36 HN -0.756 0.551
3. 36 C 37 HN 0.950 0.551
3. 37 C 38 HN 0.696 0.551

3. 38 C 39 HN -1.742 0.551
3. 39 C 40 HN -0.682 0.551
3. 40 C 41 HN 1.710 0.551
3. 41 C 42 HN -0.318 0.551
3. 42 C 43 HN 0.448 0.551
3. 43 C 44 HN 0.382 0.551
3. 44 C 45 HN 0.774 0.551
3. 45 C 46 HN -0.964 0.551
3. 46 C 47 HN -0.372 0.551
3. 49 C 50 HN -0.648 0.551
3. 50 C 51 HN 0.344 0.551
3. 51 C 52 HN -1.300 0.551
3. 52 C 53 HN -2.416 0.551
3. 53 C 54 HN 1.010 0.551
3. 54 C 55 HN -1.810 0.551
3. 56 C 57 HN 0.174 0.551
3. 57 C 58 HN 2.030 0.551
3. 58 C 59 HN -1.762 0.551
3. 59 C 60 HN 0.356 0.551
3. 60 C 61 HN 1.348 0.551
3. 61 C 62 HN 0.486 0.551
3. 62 C 63 HN 0.754 0.551
3. 3 CA 3 C 0.338 0.354
3. 7 CA 7 C -0.374 0.354
3. 8 CA 8 C 1.820 0.354
3. 9 CA 9 C 0.148 0.354
3. 10 CA 10 C 1.478 0.354
3. 11 CA 11 C -0.090 0.354
3. 12 CA 12 C 0.312 0.354
3. 14 CA 14 C -0.782 0.354
3. 15 CA 15 C -1.114 0.354
3. 16 CA 16 C -0.856 0.354
3. 17 CA 17 C -0.688 0.354
3. 18 CA 18 C -0.776 0.354
3. 19 CA 19 C 1.812 0.354
3. 20 CA 20 C -0.140 0.354
3. 21 CA 21 C -1.638 0.354
3. 22 CA 22 C -0.038 0.354
3. 23 CA 23 C 0.764 0.354
3. 24 CA 24 C -0.764 0.354
3. 25 CA 25 C -0.436 0.354
3. 26 CA 26 C -1.110 0.354
3. 27 CA 27 C 1.146 0.354
3. 28 CA 28 C 1.152 0.354
3. 29 CA 29 C -0.274 0.354
3. 30 CA 30 C -0.490 0.354
3. 31 CA 31 C 0.392 0.354
3. 32 CA 32 C 0.310 0.354
3. 33 CA 33 C -0.886 0.354
3. 34 CA 34 C 0.495 0.354
3. 35 CA 35 C 1.058 0.354
3. 36 CA 36 C -0.820 0.354
3. 38 CA 38 C 0.332 0.354
3. 39 CA 39 C 1.162 0.354
3. 40 CA 40 C -1.582 0.354
3. 41 CA 41 C 0.018 0.354
3. 42 CA 42 C -0.120 0.354
3. 43 CA 43 C 1.316 0.354
3. 44 CA 44 C -1.198 0.354
3. 46 CA 46 C -0.390 0.354
3. 47 CA 47 C -0.490 0.354
3. 48 CA 48 C -0.682 0.354
3. 49 CA 49 C -1.256 0.354
3. 50 CA 50 C 0.222 0.354
3. 51 CA 51 C -0.200 0.354
3. 52 CA 52 C 1.242 0.354
3. 53 CA 53 C -1.324 0.354
3. 56 CA 56 C -1.208 0.354
3. 57 CA 57 C -0.574 0.354
3. 58 CA 58 C 0.074 0.354
3. 59 CA 59 C 1.414 0.354
3. 60 CA 60 C -1.064 0.354
3. 61 CA 61 C 0.980 0.354
3. 62 CA 62 C -0.932 0.354
3. 63 CA 63 C 0.340 0.354
4. 4 N 4 HN 1.127 0.829
4. 5 N 5 HN -0.957 0.829
4. 7 N 7 HN 0.617 0.829

4. 8 N 8 HN -5.393 0.829
4. 9 N 9 HN -3.850 0.829
4. 10 N 10 HN -1.084 0.829
4. 11 N 11 HN 2.761 0.829
4. 12 N 12 HN 2.591 0.829
4. 13 N 13 HN 2.373 0.829
4. 15 N 15 HN 5.466 0.829
4. 16 N 16 HN 6.626 0.829
4. 17 N 17 HN 5.486 0.829
4. 19 N 19 HN -3.487 0.829
4. 20 N 20 HN -1.436 0.829
4. 21 N 21 HN 3.365 0.829
4. 22 N 22 HN -9.326 0.829
4. 23 N 23 HN -5.146 0.829
4. 24 N 24 HN 4.615 0.829
4. 25 N 25 HN 5.839 0.829
4. 26 N 26 HN 4.098 0.829
4. 27 N 27 HN -4.994 0.829
4. 28 N 28 HN 0.270 0.829
4. 29 N 29 HN 5.315 0.829
4. 30 N 30 HN 0.021 0.829
4. 31 N 31 HN -0.492 0.829
4. 32 N 32 HN -7.651 0.829
4. 33 N 33 HN -2.970 0.829
4. 34 N 34 HN -2.336 0.829
4. 35 N 35 HN -3.889 0.829
4. 36 N 36 HN -6.672 0.829
4. 37 N 37 HN 5.253 0.829
4. 38 N 38 HN 4.656 0.829
4. 39 N 39 HN -3.613 0.829
4. 40 N 40 HN 4.274 0.829
4. 41 N 41 HN 2.351 0.829
4. 42 N 42 HN -5.002 0.829
4. 43 N 43 HN 0.178 0.829
4. 44 N 44 HN -3.344 0.829
4. 45 N 45 HN -8.401 0.829
4. 46 N 46 HN -7.253 0.829
4. 47 N 47 HN -5.041 0.829
4. 48 N 48 HN -1.250 0.829
4. 49 N 49 HN -3.891 0.829
4. 50 N 50 HN -6.986 0.829
4. 51 N 51 HN 1.220 0.829
4. 52 N 52 HN -0.284 0.829
4. 53 N 53 HN -4.934 0.829
4. 54 N 54 HN -5.325 0.829
4. 58 N 58 HN -1.585 0.829
4. 59 N 59 HN 3.013 0.829
4. 60 N 60 HN -0.493 0.829
4. 61 N 61 HN 0.276 0.829
4. 62 N 62 HN 2.891 0.829
4. 63 N 63 HN -0.789 0.829
4. 64 N 64 HN 1.815 0.829
4. 3 C 4 HN -0.145 0.677
4. 4 C 5 HN 0.034 0.677
4. 5 C 6 HN -0.445 0.677
4. 6 C 7 HN -0.082 0.677
4. 7 C 8 HN -0.072 0.677
4. 8 C 9 HN 2.872 0.677
4. 9 C 10 HN -0.330 0.677
4. 10 C 11 HN 1.732 0.677
4. 11 C 12 HN -1.678 0.677
4. 12 C 13 HN -1.892 0.677
4. 14 C 15 HN -1.146 0.677
4. 15 C 16 HN -2.752 0.677
4. 16 C 17 HN -1.244 0.677
4. 17 C 18 HN 0.102 0.677
4. 18 C 19 HN -0.878 0.677
4. 19 C 20 HN -0.368 0.677
4. 20 C 21 HN -0.998 0.677
4. 22 C 23 HN 2.760 0.677
4. 23 C 24 HN -1.122 0.677
4. 24 C 25 HN -1.234 0.677
4. 25 C 26 HN -1.414 0.677
4. 27 C 28 HN 2.720 0.677
4. 28 C 29 HN -0.164 0.677
4. 29 C 30 HN -0.334 0.677
4. 30 C 31 HN -0.354 0.677
4. 31 C 32 HN -0.394 0.677

4. 32 C 33 HN 2.526 0.677
4. 33 C 34 HN 1.334 0.677
4. 34 C 35 HN 0.124 0.677
4. 35 C 36 HN 3.354 0.677
4. 36 C 37 HN -1.910 0.677
4. 37 C 38 HN -0.624 0.677
4. 38 C 39 HN 1.638 0.677
4. 39 C 40 HN -1.212 0.677
4. 40 C 41 HN -1.480 0.677
4. 41 C 42 HN 0.872 0.677
4. 42 C 43 HN -1.592 0.677
4. 43 C 44 HN -0.528 0.677
4. 44 C 45 HN 1.254 0.677
4. 45 C 46 HN 3.706 0.677
4. 46 C 47 HN -0.372 0.677
4. 47 C 48 HN 2.480 0.677
4. 48 C 49 HN -2.073 0.677
4. 49 C 50 HN 2.702 0.677
4. 51 C 52 HN -0.770 0.677
4. 52 C 53 HN 1.134 0.677
4. 53 C 54 HN -0.470 0.677
4. 54 C 55 HN 2.360 0.677
4. 56 C 57 HN -1.166 0.677
4. 57 C 58 HN 1.270 0.677
4. 58 C 59 HN -0.422 0.677
4. 59 C 60 HN 0.996 0.677
4. 60 C 61 HN -2.882 0.677
4. 61 C 62 HN 1.346 0.677
4. 62 C 63 HN -1.086 0.677
4. 3 CA 3 C 0.598 0.260
4. 4 CA 4 C -0.336 0.260
4. 5 CA 5 C 0.616 0.260
4. 6 CA 6 C -1.064 0.260
4. 7 CA 7 C 1.406 0.260
4. 8 CA 8 C -1.520 0.260
4. 9 CA 9 C 0.828 0.260
4. 10 CA 10 C -0.312 0.260
4. 11 CA 11 C -0.430 0.260
4. 14 CA 14 C -0.462 0.260
4. 16 CA 16 C -0.676 0.260
4. 17 CA 17 C 1.002 0.260
4. 18 CA 18 C -0.846 0.260
4. 19 CA 19 C -0.528 0.260
4. 20 CA 20 C 1.500 0.260
4. 21 CA 21 C 0.192 0.260
4. 22 CA 22 C -1.098 0.260
4. 23 CA 23 C -0.226 0.260
4. 24 CA 24 C -0.094 0.260
4. 25 CA 25 C -0.676 0.260
4. 26 CA 26 C 1.310 0.260
4. 27 CA 27 C -0.574 0.260
4. 28 CA 28 C -0.808 0.260
4. 29 CA 29 C 1.276 0.260
4. 30 CA 30 C -0.680 0.260
4. 32 CA 32 C -0.550 0.260
4. 33 CA 33 C -0.916 0.260
4. 34 CA 34 C -0.465 0.260
4. 35 CA 35 C -0.952 0.260
4. 36 CA 36 C -0.730 0.260
4. 37 CA 37 C 0.694 0.260
4. 38 CA 38 C -0.458 0.260
4. 39 CA 39 C -0.788 0.260
4. 40 CA 40 C 0.028 0.260
4. 41 CA 41 C -0.202 0.260
4. 42 CA 42 C -0.140 0.260
4. 43 CA 43 C -0.534 0.260
4. 44 CA 44 C -0.168 0.260
4. 45 CA 45 C -1.160 0.260
4. 46 CA 46 C 0.320 0.260
4. 47 CA 47 C -0.610 0.260
4. 48 CA 48 C 0.487 0.260
4. 49 CA 49 C -0.166 0.260
4. 50 CA 50 C -1.078 0.260
4. 51 CA 51 C 0.820 0.260
4. 52 CA 52 C -1.068 0.260
4. 53 CA 53 C -0.034 0.260
4. 54 CA 54 C -0.624 0.260
4. 57 CA 57 C -0.824 0.260

4. 58 CA 58 C 0.214 0.260
4. 60 CA 60 C 1.006 0.260
4. 61 CA 61 C -0.970 0.260
4. 62 CA 62 C -0.472 0.260
4. 63 CA 63 C 0.550 0.260
5. 8 N 8 HN 5.137 0.707
5. 9 N 9 HN -2.351 0.707
5. 11 N 11 HN -4.315 0.707
5. 12 N 12 HN 4.253 0.707
5. 13 N 13 HN -19.890 0.707
5. 15 N 15 HN -5.903 0.707
5. 16 N 16 HN -11.392 0.707
5. 17 N 17 HN 3.308 0.707
5. 18 N 18 HN 18.004 0.707
5. 19 N 19 HN 13.250 0.707
5. 20 N 20 HN 10.277 0.707
5. 21 N 21 HN 5.954 0.707
5. 22 N 22 HN 19.813 0.707
5. 23 N 23 HN 8.156 0.707
5. 24 N 24 HN -15.195 0.707
5. 25 N 25 HN -6.888 0.707
5. 26 N 26 HN -5.917 0.707
5. 28 N 28 HN 9.469 0.707
5. 29 N 29 HN 6.628 0.707
5. 30 N 30 HN 4.278 0.707
5. 31 N 31 HN -10.859 0.707
5. 32 N 32 HN 10.721 0.707
5. 33 N 33 HN 13.849 0.707
5. 35 N 35 HN 5.946 0.707
5. 37 N 37 HN -10.327 0.707
5. 39 N 39 HN 4.989 0.707
5. 40 N 40 HN 8.717 0.707
5. 41 N 41 HN 9.421 0.707
5. 42 N 42 HN 14.004 0.707
5. 43 N 43 HN 4.275 0.707
5. 44 N 44 HN 7.993 0.707
5. 45 N 45 HN 20.790 0.707
5. 46 N 46 HN 19.365 0.707
5. 47 N 47 HN 12.308 0.707
5. 48 N 48 HN 3.364 0.707
5. 49 N 49 HN 14.213 0.707
5. 50 N 50 HN 13.348 0.707
5. 51 N 51 HN -16.870 0.707
5. 52 N 52 HN -10.485 0.707
5. 53 N 53 HN 13.401 0.707
5. 54 N 54 HN 15.692 0.707
5. 55 N 55 HN 16.625 0.707
5. 57 N 57 HN 6.862 0.707
5. 58 N 58 HN 13.923 0.707
5. 59 N 59 HN -20.360 0.707
5. 60 N 60 HN 9.321 0.707
5. 61 N 61 HN 7.801 0.707
5. 62 N 62 HN 1.441 0.707
5. 63 N 63 HN -16.159 0.707
5. 6 C 7 HN -2.582 0.396
5. 7 C 8 HN 5.888 0.396
5. 9 C 10 HN 7.400 0.396
5. 10 C 11 HN -2.888 0.396
5. 11 C 12 HN -2.518 0.396
5. 12 C 13 HN 5.028 0.396
5. 14 C 15 HN 8.384 0.396
5. 15 C 16 HN -2.302 0.396
5. 16 C 17 HN 4.936 0.396
5. 17 C 18 HN -2.998 0.396
5. 18 C 19 HN -3.658 0.396
5. 19 C 20 HN 4.382 0.396
5. 20 C 21 HN -0.568 0.396
5. 21 C 22 HN -2.316 0.396
5. 22 C 23 HN -3.060 0.396
5. 23 C 24 HN -0.992 0.396
5. 24 C 25 HN 8.756 0.396
5. 25 C 26 HN -3.474 0.396
5. 26 C 27 HN 5.932 0.396
5. 27 C 28 HN -5.380 0.396
5. 28 C 29 HN -3.464 0.396
5. 29 C 30 HN 4.176 0.396
5. 30 C 31 HN -1.414 0.396
5. 31 C 32 HN 6.086 0.396

5. 32 C 33 HN -4.734 0.396
5. 34 C 35 HN 0.854 0.396
5. 36 C 37 HN 8.260 0.396
5. 37 C 38 HN 2.446 0.396
5. 38 C 39 HN -1.662 0.396
5. 39 C 40 HN -1.492 0.396
5. 40 C 41 HN -0.290 0.396
5. 41 C 42 HN -4.678 0.396
5. 42 C 43 HN 5.948 0.396
5. 43 C 44 HN -2.868 0.396
5. 44 C 45 HN -1.816 0.396
5. 46 C 47 HN -3.612 0.396
5. 50 C 51 HN 7.994 0.396
5. 51 C 52 HN 3.200 0.396
5. 52 C 53 HN 0.854 0.396
5. 54 C 55 HN -3.570 0.396
5. 56 C 57 HN -3.566 0.396
5. 57 C 58 HN -3.420 0.396
5. 58 C 59 HN 7.678 0.396
5. 59 C 60 HN -4.344 0.396
5. 60 C 61 HN -2.192 0.396
5. 61 C 62 HN -4.234 0.396
5. 62 C 63 HN 6.254 0.396
5. 63 C 64 HN 2.984 0.396
5. 7 CA 7 C -2.844 0.296
5. 8 CA 8 C 4.580 0.296
5. 9 CA 9 C -1.632 0.296
5. 11 CA 11 C 5.220 0.296
5. 12 CA 12 C -3.238 0.296
5. 14 CA 14 C -1.132 0.296
5. 15 CA 15 C 1.286 0.296
5. 16 CA 16 C 0.404 0.296
5. 17 CA 17 C -2.848 0.296
5. 18 CA 18 C 4.384 0.296
5. 19 CA 19 C -0.168 0.296
5. 20 CA 20 C -3.760 0.296
5. 21 CA 21 C 2.572 0.296
5. 22 CA 22 C -1.778 0.296
5. 23 CA 23 C 2.654 0.296
5. 24 CA 24 C -1.834 0.296
5. 25 CA 25 C 5.234 0.296
5. 26 CA 26 C -2.620 0.296
5. 27 CA 27 C -1.184 0.296
5. 28 CA 28 C 5.622 0.296
5. 29 CA 29 C -2.834 0.296
5. 30 CA 30 C 3.170 0.296
5. 31 CA 31 C -2.138 0.296
5. 32 CA 32 C -1.190 0.296
5. 33 CA 33 C 3.924 0.296
5. 34 CA 34 C -1.985 0.296
5. 35 CA 35 C 4.158 0.296
5. 36 CA 36 C -1.940 0.296
5. 37 CA 37 C -0.316 0.296
5. 38 CA 38 C -1.588 0.296
5. 39 CA 39 C 2.212 0.296
5. 40 CA 40 C 1.938 0.296
5. 41 CA 41 C -0.502 0.296
5. 42 CA 42 C -0.200 0.296
5. 43 CA 43 C -0.554 0.296
5. 44 CA 44 C 1.462 0.296
5. 45 CA 45 C -2.150 0.296
5. 46 CA 46 C -0.120 0.296
5. 47 CA 47 C 0.680 0.296
5. 48 CA 48 C 0.447 0.296
5. 49 CA 49 C 3.494 0.296
5. 50 CA 50 C -2.288 0.296
5. 51 CA 51 C -1.370 0.296
5. 52 CA 52 C -0.958 0.296
5. 53 CA 53 C 1.766 0.296
5. 54 CA 54 C 3.376 0.296
5. 56 CA 56 C -2.878 0.296
5. 57 CA 57 C 4.686 0.296
5. 58 CA 58 C -1.896 0.296
5. 59 CA 59 C -1.816 0.296
5. 60 CA 60 C -3.224 0.296
5. 61 CA 61 C 2.300 0.296
5. 62 CA 62 C -1.572 0.296
5. 63 CA 63 C -1.230 0.296

6. 4 N 4 HN 0.466 0.570
6. 5 N 5 HN 2.173 0.570
6. 6 N 6 HN -0.842 0.570
6. 8 N 8 HN 4.427 0.570
6. 9 N 9 HN 6.823 0.570
6. 10 N 10 HN 4.575 0.570
6. 11 N 11 HN 1.629 0.570
6. 12 N 12 HN 3.420 0.570
6. 13 N 13 HN 2.400 0.570
6. 15 N 15 HN -4.399 0.570
6. 16 N 16 HN -3.883 0.570
6. 17 N 17 HN -5.278 0.570
6. 19 N 19 HN -1.524 0.570
6. 20 N 20 HN 0.414 0.570
6. 21 N 21 HN -8.847 0.570
6. 22 N 22 HN -0.926 0.570
6. 23 N 23 HN 5.535 0.570
6. 24 N 24 HN -3.840 0.570
6. 25 N 25 HN -7.613 0.570
6. 26 N 26 HN -8.635 0.570
6. 27 N 27 HN -2.476 0.570
6. 28 N 28 HN 2.861 0.570
6. 29 N 29 HN -6.889 0.570
6. 30 N 30 HN 4.036 0.570
6. 31 N 31 HN 6.184 0.570
6. 32 N 32 HN 4.530 0.570
6. 34 N 34 HN -9.829 0.570
6. 35 N 35 HN -2.889 0.570
6. 36 N 36 HN -4.942 0.570
6. 37 N 37 HN -5.193 0.570
6. 38 N 38 HN 1.022 0.570
6. 39 N 39 HN 3.710 0.570
6. 40 N 40 HN -3.926 0.570
6. 41 N 41 HN -2.369 0.570
6. 43 N 43 HN 3.382 0.570
6. 44 N 44 HN 0.300 0.570
6. 45 N 45 HN -1.584 0.570
6. 46 N 46 HN -3.735 0.570
6. 47 N 47 HN 1.392 0.570
6. 48 N 48 HN 2.363 0.570
6. 49 N 49 HN 0.596 0.570
6. 50 N 50 HN 2.425 0.570
6. 51 N 51 HN 5.732 0.570
6. 52 N 52 HN 5.902 0.570
6. 53 N 53 HN 0.588 0.570
6. 54 N 54 HN -0.331 0.570
6. 57 N 57 HN 0.629 0.570
6. 58 N 58 HN -3.007 0.570
6. 59 N 59 HN 4.886 0.570
6. 60 N 60 HN 3.224 0.570
6. 61 N 61 HN 3.277 0.570
6. 62 N 62 HN 1.975 0.570
6. 63 N 63 HN 7.138 0.570
6. 64 N 64 HN -0.037 0.570
6. 3 C 4 HN 1.005 0.233
6. 4 C 5 HN -0.266 0.233
6. 5 C 6 HN -1.645 0.233
6. 7 C 8 HN -2.212 0.233
6. 8 C 9 HN -0.318 0.233
6. 9 C 10 HN -1.500 0.233
6. 10 C 11 HN -1.938 0.233
6. 11 C 12 HN 1.112 0.233
6. 12 C 13 HN -2.462 0.233
6. 15 C 16 HN 3.038 0.233
6. 16 C 17 HN 2.026 0.233
6. 17 C 18 HN -1.528 0.233
6. 18 C 19 HN 2.532 0.233
6. 19 C 20 HN -2.198 0.233
6. 20 C 21 HN 0.582 0.233
6. 21 C 22 HN 1.414 0.233
6. 22 C 23 HN -1.500 0.233
6. 23 C 24 HN 0.268 0.233
6. 24 C 25 HN 0.166 0.233
6. 25 C 26 HN 2.156 0.233
6. 26 C 27 HN 0.052 0.233
6. 27 C 28 HN -1.250 0.233
6. 28 C 29 HN 0.466 0.233
6. 29 C 30 HN 1.016 0.233

6. 30 C 31 HN -1.364 0.233
6. 31 C 32 HN -2.564 0.233
6. 32 C 33 HN -0.914 0.233
6. 34 C 35 HN 2.654 0.233
6. 35 C 36 HN -0.786 0.233
6. 36 C 37 HN 0.070 0.233
6. 37 C 38 HN 1.736 0.233
6. 38 C 39 HN -1.912 0.233
6. 39 C 40 HN -0.942 0.233
6. 40 C 41 HN 0.460 0.233
6. 42 C 43 HN -0.632 0.233
6. 43 C 44 HN -0.958 0.233
6. 44 C 45 HN 3.104 0.233
6. 45 C 46 HN -1.154 0.233
6. 46 C 47 HN 1.258 0.233
6. 47 C 48 HN -0.640 0.233
6. 48 C 49 HN 1.197 0.233
6. 49 C 50 HN 0.002 0.233
6. 50 C 51 HN -0.646 0.233
6. 51 C 52 HN -1.370 0.233
6. 52 C 53 HN -2.526 0.233
6. 53 C 54 HN 3.100 0.233
6. 54 C 55 HN -1.570 0.233
6. 56 C 57 HN -1.196 0.233
6. 57 C 58 HN 3.220 0.233
6. 58 C 59 HN -2.332 0.233
6. 59 C 60 HN -0.764 0.233
6. 60 C 61 HN 1.028 0.233
6. 61 C 62 HN -1.174 0.233
6. 62 C 63 HN -1.136 0.233
6. 63 C 64 HN -1.736 0.233
6. 3 CA 3 C -0.082 0.173
6. 4 CA 4 C -0.316 0.173
6. 5 CA 5 C 0.726 0.173
6. 6 CA 6 C 0.186 0.173
6. 7 CA 7 C 0.236 0.173
6. 8 CA 8 C 0.620 0.173
6. 9 CA 9 C -0.412 0.173
6. 10 CA 10 C 2.458 0.173
6. 11 CA 11 C -0.410 0.173
6. 12 CA 12 C 1.132 0.173
6. 14 CA 14 C -0.372 0.173
6. 15 CA 15 C -1.884 0.173
6. 16 CA 16 C -0.616 0.173
6. 18 CA 18 C -1.216 0.173
6. 19 CA 19 C 2.002 0.173
6. 20 CA 20 C 0.600 0.173
6. 21 CA 21 C -1.258 0.173
6. 22 CA 22 C -0.188 0.173
6. 23 CA 23 C 0.644 0.173
6. 24 CA 24 C 0.356 0.173
6. 25 CA 25 C -0.556 0.173
6. 26 CA 26 C -0.740 0.173
6. 27 CA 27 C 1.926 0.173
6. 28 CA 28 C 0.032 0.173
6. 29 CA 29 C -0.104 0.173
6. 30 CA 30 C -1.250 0.173
6. 31 CA 31 C 0.872 0.173
6. 32 CA 32 C -0.120 0.173
6. 33 CA 33 C -0.986 0.173
6. 34 CA 34 C -0.225 0.173
6. 35 CA 35 C 0.518 0.173
6. 36 CA 36 C 0.370 0.173
6. 37 CA 37 C -0.926 0.173
6. 38 CA 38 C 0.572 0.173
6. 39 CA 39 C 1.372 0.173
6. 40 CA 40 C -1.232 0.173
6. 41 CA 41 C -0.502 0.173
6. 42 CA 42 C -0.960 0.173
6. 43 CA 43 C 1.786 0.173
6. 44 CA 44 C -1.508 0.173
6. 45 CA 45 C 1.110 0.173
6. 46 CA 46 C -1.370 0.173
6. 47 CA 47 C -0.950 0.173
6. 48 CA 48 C -1.322 0.173
6. 50 CA 50 C 0.752 0.173
6. 51 CA 51 C -1.210 0.173
6. 52 CA 52 C 1.972 0.173

6. 53 CA 53 C -1.244 0.173
6. 54 CA 54 C -0.414 0.173
6. 56 CA 56 C 0.442 0.173
6. 57 CA 57 C -1.024 0.173
6. 58 CA 58 C -0.776 0.173
6. 59 CA 59 C 1.954 0.173
6. 60 CA 60 C -0.674 0.173
6. 61 CA 61 C 1.600 0.173
6. 62 CA 62 C 0.008 0.173
7. 5 N 5 HN 0.957 0.496
7. 6 N 6 HN -1.117 0.496
7. 7 N 7 HN 6.179 0.496
7. 8 N 8 HN 8.617 0.496
7. 9 N 9 HN 7.457 0.496
7. 10 N 10 HN 3.099 0.496
7. 11 N 11 HN -3.052 0.496
7. 12 N 12 HN -1.319 0.496
7. 13 N 13 HN 0.879 0.496
7. 15 N 15 HN -8.047 0.496
7. 16 N 16 HN -6.295 0.496
7. 17 N 17 HN -6.659 0.496
7. 18 N 18 HN 6.031 0.496
7. 19 N 19 HN 3.559 0.496
7. 20 N 20 HN 3.664 0.496
7. 21 N 21 HN -7.135 0.496
7. 22 N 22 HN 3.937 0.496
7. 23 N 23 HN 3.795 0.496
7. 24 N 24 HN -6.764 0.496
7. 25 N 25 HN -8.673 0.496
7. 26 N 26 HN -8.230 0.496
7. 27 N 27 HN -3.007 0.496
7. 28 N 28 HN -0.937 0.496
7. 29 N 29 HN -6.737 0.496
7. 30 N 30 HN -0.362 0.496
7. 31 N 31 HN 5.192 0.496
7. 32 N 32 HN 8.258 0.496
7. 33 N 33 HN 4.402 0.496
7. 35 N 35 HN -2.868 0.496
7. 36 N 36 HN -4.523 0.496
7. 37 N 37 HN -6.830 0.496
7. 38 N 38 HN -0.694 0.496
7. 39 N 39 HN 7.343 0.496
7. 40 N 40 HN -5.160 0.496
7. 41 N 41 HN -3.603 0.496
7. 42 N 42 HN -0.912 0.496
7. 43 N 43 HN -1.052 0.496
7. 44 N 44 HN -0.027 0.496
7. 45 N 45 HN 2.741 0.496
7. 46 N 46 HN 2.542 0.496
7. 47 N 47 HN 6.674 0.496
7. 48 N 48 HN 6.979 0.496
7. 50 N 50 HN 6.429 0.496
7. 51 N 51 HN 5.022 0.496
7. 52 N 52 HN 7.670 0.496
7. 53 N 53 HN 5.815 0.496
7. 54 N 54 HN 6.090 0.496
7. 55 N 55 HN -0.888 0.496
7. 57 N 57 HN -2.497 0.496
7. 58 N 58 HN 0.642 0.496
7. 60 N 60 HN -1.640 0.496
7. 61 N 61 HN -1.235 0.496
7. 62 N 62 HN -3.005 0.496
7. 63 N 63 HN 7.024 0.496
7. 64 N 64 HN -0.881 0.496
7. 4 C 5 HN 0.424 0.509
7. 5 C 6 HN -0.495 0.509
7. 6 C 7 HN -0.292 0.509
7. 7 C 8 HN -2.972 0.509
7. 8 C 9 HN -2.518 0.509
7. 9 C 10 HN -1.530 0.509
7. 10 C 11 HN -2.058 0.509
7. 11 C 12 HN 2.122 0.509
7. 14 C 15 HN 1.524 0.509
7. 15 C 16 HN 3.218 0.509
7. 16 C 17 HN 1.756 0.509
7. 17 C 18 HN 1.102 0.509
7. 18 C 19 HN 0.982 0.509
7. 20 C 21 HN 1.502 0.509

7. 21 C 22 HN 0.524 0.509
7. 22 C 23 HN -2.640 0.509
7. 23 C 24 HN 1.468 0.509
7. 24 C 25 HN -0.234 0.509
7. 25 C 26 HN 1.816 0.509
7. 26 C 27 HN 1.552 0.509
7. 27 C 28 HN -0.950 0.509
7. 28 C 29 HN 0.046 0.509
7. 29 C 30 HN 1.946 0.509
7. 30 C 31 HN -0.084 0.509
7. 31 C 32 HN -0.984 0.509
7. 33 C 34 HN 1.514 0.509
7. 34 C 35 HN 3.114 0.509
7. 36 C 37 HN 0.850 0.509
7. 37 C 38 HN 2.046 0.509
7. 38 C 39 HN -0.752 0.509
7. 39 C 40 HN 0.238 0.509
7. 40 C 41 HN 1.440 0.509
7. 41 C 42 HN -0.728 0.509
7. 42 C 43 HN 0.228 0.509
7. 43 C 44 HN -0.118 0.509
7. 44 C 45 HN 1.484 0.509
7. 45 C 46 HN -1.614 0.509
7. 47 C 48 HN -1.940 0.509
7. 49 C 50 HN -0.088 0.509
7. 50 C 51 HN -0.206 0.509
7. 51 C 52 HN -0.800 0.509
7. 52 C 53 HN -2.776 0.509
7. 53 C 54 HN 1.430 0.509
7. 54 C 55 HN -2.150 0.509
7. 56 C 57 HN 0.674 0.509
7. 57 C 58 HN 3.150 0.509
7. 58 C 59 HN -1.732 0.509
7. 59 C 60 HN -1.064 0.509
7. 60 C 61 HN 1.918 0.509
7. 61 C 62 HN -0.044 0.509
7. 62 C 63 HN 0.504 0.509
7. 63 C 64 HN -1.336 0.509
7. 3 CA 3 C -0.502 0.222
7. 4 CA 4 C 0.564 0.222
7. 7 CA 7 C -0.064 0.222
7. 9 CA 9 C 0.138 0.222
7. 10 CA 10 C 1.948 0.222
7. 11 CA 11 C -0.270 0.222
7. 12 CA 12 C 0.792 0.222
7. 14 CA 14 C -0.002 0.222
7. 15 CA 15 C -2.244 0.222
7. 16 CA 16 C -0.126 0.222
7. 17 CA 17 C -0.268 0.222
7. 18 CA 18 C -0.796 0.222
7. 19 CA 19 C 1.962 0.222
7. 20 CA 20 C -0.470 0.222
7. 21 CA 21 C -1.398 0.222
7. 22 CA 22 C 0.752 0.222
7. 23 CA 23 C 0.574 0.222
7. 24 CA 24 C -0.014 0.222
7. 25 CA 25 C -0.206 0.222
7. 26 CA 26 C -1.980 0.222
7. 27 CA 27 C 1.476 0.222
7. 28 CA 28 C 1.192 0.222
7. 29 CA 29 C 0.676 0.222
7. 30 CA 30 C -1.330 0.222
7. 31 CA 31 C 0.402 0.222
7. 33 CA 33 C -0.636 0.222
7. 34 CA 34 C 0.495 0.222
7. 35 CA 35 C 0.868 0.222
7. 36 CA 36 C 0.330 0.222
7. 37 CA 37 C -1.396 0.222
7. 38 CA 38 C 0.712 0.222
7. 39 CA 39 C 1.342 0.222
7. 40 CA 40 C -1.442 0.222
7. 41 CA 41 C -0.052 0.222
7. 42 CA 42 C -0.200 0.222
7. 43 CA 43 C 1.486 0.222
7. 44 CA 44 C -1.058 0.222
7. 45 CA 45 C 1.410 0.222
7. 46 CA 46 C -0.590 0.222
7. 47 CA 47 C 0.160 0.222

7. 49 CA 49 C -1.846 0.222
7. 50 CA 50 C 0.352 0.222
7. 51 CA 51 C -0.500 0.222
7. 52 CA 52 C 1.492 0.222
7. 53 CA 53 C -1.154 0.222
7. 54 CA 54 C -0.444 0.222
7. 56 CA 56 C -0.668 0.222
7. 57 CA 57 C -0.724 0.222
7. 58 CA 58 C -0.066 0.222
7. 59 CA 59 C 1.384 0.222
7. 60 CA 60 C -1.404 0.222
7. 61 CA 61 C 1.970 0.222
7. 62 CA 62 C -0.432 0.222
7. 63 CA 63 C 1.230 0.222
